# Cell-Penetrating Peptides in Diagnosis and Treatment of Human Diseases: From Preclinical Research to Clinical Application

**DOI:** 10.3389/fphar.2020.00697

**Published:** 2020-05-20

**Authors:** Jing Xie, Ye Bi, Huan Zhang, Shiyan Dong, Lesheng Teng, Robert J. Lee, Zhaogang Yang

**Affiliations:** ^1^School of Pharmacy and Bioengineering, Chongqing University of Technology, Chongqing, China; ^2^Practice Training Center, Changchun University of Chinese Medicine, Changchun, China; ^3^School of Life Sciences, Jilin University, Changchun, China; ^4^Division of Pharmaceutics and Pharmacology, The Ohio State University, Columbus, OH, United States; ^5^Department of Radiation Oncology, University of Texas Southwestern Medical Center, Dallas, TX, United States

**Keywords:** cell-penetrating peptides, cellular uptake, diagnosis, translocate, targeting

## Abstract

Cell-penetrating peptides (CPPs) are short peptides (fewer than 30 amino acids) that have been predominantly used in basic and preclinical research during the last 30 years. Since they are not only capable of translocating themselves into cells but also facilitate drug or CPP/cargo complexes to translocate across the plasma membrane, they have potential applications in the disease diagnosis and therapy, including cancer, inflammation, central nervous system disorders, otologic and ocular disorders, and diabetes. However, no CPPs or CPP/cargo complexes have been approved by the US Food and Drug Administration (FDA). Many issues should be addressed before translating CPPs into clinics. In this review, we summarize recent developments and innovations in preclinical studies and clinical trials based on using CPP for improved delivery, which have revealed that CPPs or CPP-based delivery systems present outstanding diagnostic therapeutic delivery potential.

## Introduction

Successful systemic administration of a drug usually includes a series of steps such as a long circulation, penetration of a biological barrier, uptake in recipient cells, and endosomal escape to the cytosolic space after endocytosis, each of which has its own set of constraints. In fact, a broad range of bioactive molecules have difficulty accessing the target, and penetrate the cell membrane to achieve the therapeutic effect. Because plasma membranes work as effective biochemical barriers, they play a critical role in preventing exogenous invasion (Groves, 2019). For example, peptides and oligonucleotides have been extensively evaluated in various therapeutic studies, which tend to have relatively low transmembrane efficiency, and therefore, achieving desired drug concentrations at the therapeutic site is challenging. Current strategies for delivery of macromolecules, such as nanoparticles, liposomes, viral-based vectors, microinjection, and electroporation (Kang et al., 2016; Shi et al., 2018; Hao et al., 2019; Yang et al., 2019b; Yang et al., 2019c), may result in high toxicity, poor specificity, immunogenicity, as well as low delivery efficiency and efficacy (Swain et al., 2016). Accordingly, an approach for the delivery of macromolecules into target cells with high efficiency and efficacy is urgently needed.

Generally, it is believed that hydrophilic macromolecules can only be internalized by the classical endocytosis pathway. However, several peptides with membrane penetrating function could transport hydrophilic macromolecules to eukaryotic cells through energy-independent pathways (Lindgren et al., 2000; Gupta et al., 2005). They are termed as cell-penetrating peptides (CPPs), also known as protein transduction domains (PTDs), which are short peptides (no more than 30 residues) that have been predominantly evaluated in basic and preclinical research for the disease diagnosis and therapy including cancer, inflammation, central nervous system disorders, otoprotective, ocular disorders, and diabetes. They are not only able to translocate small macromolecular drugs, nucleic acids (Tobin et al., 1991), proteins, viruses, imaging agents across plasma membranes but also allow CPP/cargo complexes to transport across the cell membrane, by different endocytosis pathways depending on the types of CPPs (Tripathi et al., 2018). Different from other delivery strategies mentioned above, CPPs can enter the cells in a noninvasive way, as they usually do not disturb the structure of the plasma membranes and are considered safe and highly efficient. CPPs was first introduced by two research groups in 1988 (Frankel and Pabo, 1988; Green and Loewenstein, 1988). Both Frankel et al. and Green et al. observed that the HIV-trans-activator of transcription (TAT) protein could enter tissue-cultured cells, target into cell nucleus, and finally result in target gene expression. In 1991, Joliot et al. revealed that the homeodomain of Antennapedia (pAntp), a synthetic peptide with 60 amino acids long, was internalized by nerve cells (Joliot et al., 1991). Subsequently, Derossi et al. found a short peptide with 16 amino acids from the third helix of the antennapedia homeodomain (RQIKIYFQNRRMKWKK), named penetratin, was able to penetrate the plasma membrane (Derossi et al., 1994). From then on, various CPPs have been characterized from natural sources and synthetic sources (Tru Van et al., 2018).

CPPs have been successfully applied in the delivery of different types of drugs, nanoparticles, and liposomes for disease diagnosis and treatment. Although the number of CPP-based clinical trials has been greatly increased, in fact, no CPPs or CPP/cargo complexes have been approved by the US Food and Drug Administration (FDA). In this review, we will systematically summarize the latest application strategies of CPPs in various diseases diagnosis and treatment over the last 5 years and emphasize the preclinical research and clinical application of CPPs. We present a classification of CPPs and explain cellular uptake mechanisms of these peptides. We will discuss the deficiency and limitation of CPPs in clinical applications. Answering these questions will allow us to develop a more effective drug delivery system. Finally, we will discuss various new strategies for the application of CPPs in different disease diagnosis and therapy.

## Classification of CPPs

**Figure 1:**
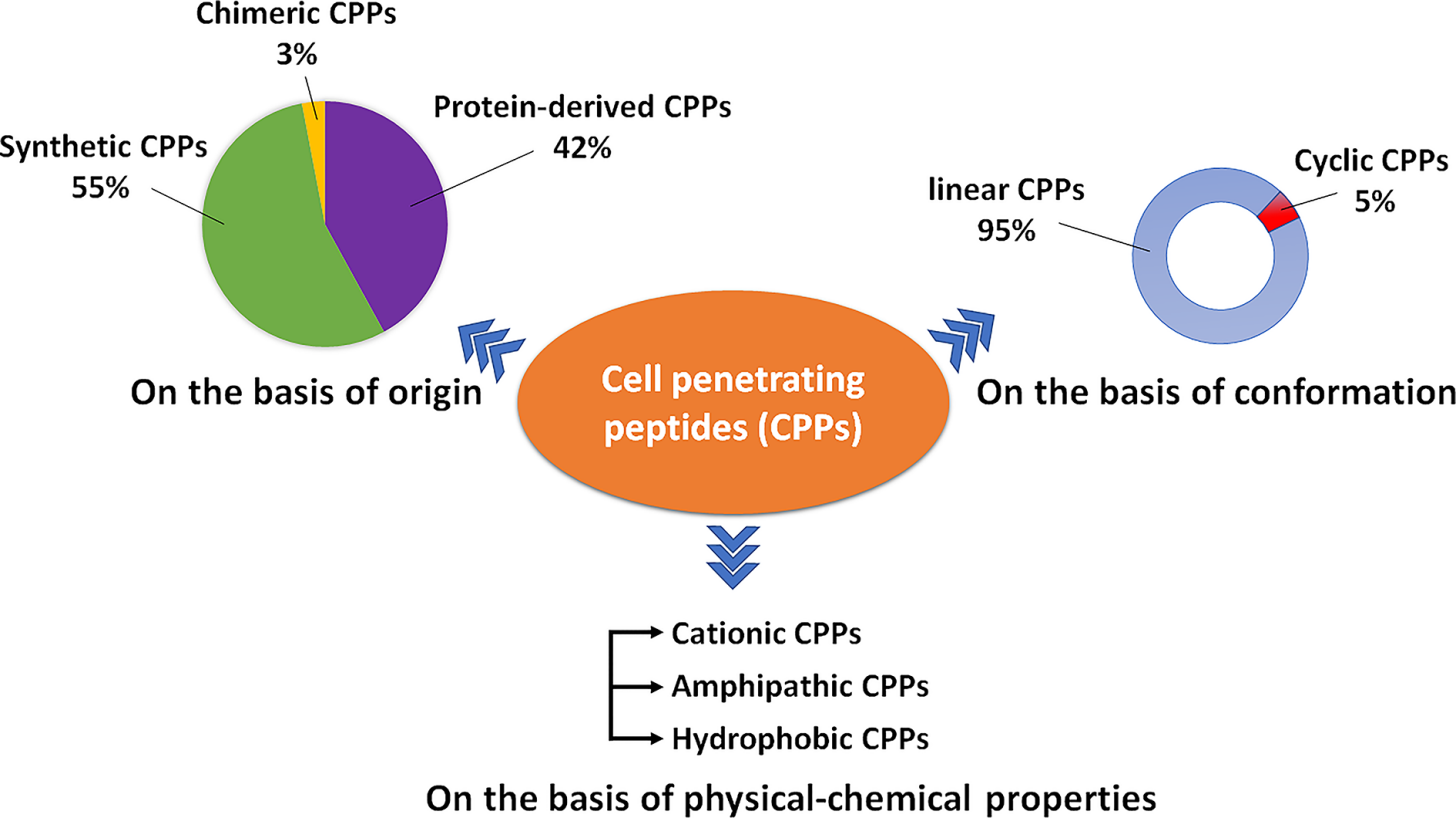
A schematic diagram illustrating the types of CPPs.

Currently, CPPsite 2.0 (http://crdd.osdd.net/raghava/cppsite/) database contains about 1850 kinds of CPP sequences, and the number is expected to continue to increase (Agrawal et al., 2016). CPPs are highly diverse in physicochemical and biological properties, different types of CPPs have different length, charge, solubility, and hydrophobicity (Kauffman et al., 2015). At present, CPP is often classified according to different characteristics, as shown in **Figure 1** and **Table 1**, including their origin, conformation, and physical and chemical properties.

**Table 1:** Classification of the types of CPPs.

Peptide	Sequence	Length	Origin	References
Cationic CPPs				
TAT	RKKRRQRRR	9	Protein derived	(Baoum et al., 2012)
R8	RRRRRRRR	8	Synthetic	(Chu et al., 2015)
DPV3	RKKRRRESRKKRRRES	16	Protein derived	(De coupade et al., 2005)
DPV6	GRPRESGKKRKRKRLKP	17	Protein derived	(De coupade et al., 2005)
Penetratin	RQIKIWFQNRRMKWKK	16	Protein derived	(Nielsen et al., 2014)
R9-TAT	GRRRRRRRRRPPQ	13	Protein derived	(Futaki et al., 2001)
				
Amphipathic CPPs				
pVEC	LLIILRRRIRKQAHAHSK	18	Protein derived	(Eggimann et al., 2014)
ARF (19-31)	RVRVFVVHIPRLT	13	Protein derived	(Johansson et al., 2008)
MPG	GALFLGFLGAAGSTMGAWSQPKKKRKV	27	Chimeric	(Simeoni, 2003)
MAP	KLALKLALKALKAALKLA	18	Synthetic	(Wada et al., 2013)
Transportan	GWTLNSAGYLLGKINLKALAALAKKIL	27	Protein derived	(Pae et al., 2014)
				
Hydrophobic CPPs				
Bip4	VSALK	5	Protein derived	(Gomez et al., 2010)
C105Y	CSIPPEVKFNPFVYLI	16	Protein derived	(Rhee and Davis, 2006)
Melittin	GIGAVLKVLTTGLPALISWIKRKRQQ	26	Protein derived	(Hou et al., 2013)
gH625	HGLASTLTRWAHYNALIRAF	20	Protein derived	(Galdiero et al., 2015)

### Classification Based on Origin

Based on their origins, CPPs can be divided into protein-derived CPPs, synthetic CPPs, and chimeric CPPs. (1). Protein-derived CPPs, including the TAT protein (Vives et al., 1997) and penetratin (Derossi et al., 1994), can enter the cell because they have a specific motifs or helical structures (Traub, 2009). (2). Synthetic CPPs. In this group, polyarginine 8-10-mers are most widely studied because of their high efficiency in cellular uptake (Mitchell et al., 2000). (3). Chimeric CPPs. This group of CPPs is generally recognized as a transition from natural to synthetic CPPs since they contain sequences from two or more different naturally occurring proteins. Examples include amphipathic peptide (CADY) (20 amino acids) which combines aromatic residue (Tryptophan, W) and cationic residue (Arginine, R) (Crombez et al., 2009).

### Classification Based on Conformation

Depending on their conformation, CPPs are divided into linear CPPs and cyclic CPPs. Many current studies have confirmed that cyclic CPPs offer advantages compared to their linear counterparts. Compared with linear CPPs, cyclic CPPs have higher cell permeability and higher affinity with the target receptor on the cell, so the transmembrane ability is further increased through receptor-mediated uptake (Dougherty et al., 2019). In addition, linear CPPs are usually sensitive to proteolytic hydrolysis, which results in poor pharmacokinetic properties *in vivo* (Bechara and Sagan, 2013), while cyclic CPPs generally have a higher resistance to proteolysis (Qian et al., 2016). At the same time, some cyclic CPPs can be taken up without endosome degradation and have the characteristics of targeting the nucleus (Mandal et al., 2011).

### Classification Based on Physical–Chemical Character

Based on differences in physicochemical characters, CPPs can be classified into three subgroups: cationic CPPs, amphipathic CPPs, and hydrophobic CPPs. Under normal physiological pH conditions, the positive charge of cationic CPPs shows excellent affinity with the cytoplasmic membrane. The cationic CPPs combine with the cell membrane glycoprotein which is negatively charged through electrostatic interaction and then internalizes into the cell through a mechanism independent of the receptor. The key factors affecting the activity of cationic CPPs are the number and position of positively charged arginines in the CPP structure (Xu et al., 2019). Most cationic CPPs usually contain more than five positively charged amino acids (Borrelli et al., 2018). The poly-arginine stretches have the highest cell uptake capacity and have therapeutic potential. The results of the study show that the internalization capacity of oligoarginine increases with its length (Chu et al., 2015), but for delivery purposes, the optimal length is R8 to R10. Higher values will have irreversible side effects on the cells and reduce overall delivery efficiency (Verdurmen and Brock, 2011). Nuclear localization signal (NLS) is a small peptide rich in arginine, lysine or proline commonly found in CPP. NLS can be transported into the cell nucleus through the classical nuclear introduction pathway (Tammam et al., 2017). Due to the limited positive charge and the limited membrane penetration ability of NLS, it is often combined with hydrophobic or amphoteric amino acid sequences to produce effective and versatile amphiphilic vectors including MPG (Lee et al., 2014) and Pep-1 (Yang et al., 2005).

Among the CPPs currently found, amphipathic CPPs are the most common, accounting for more than 40%. Amphiphilic CPPs contain polar and non-polar amino acid regions, and the non-polar regions are rich in hydrophobic amino acids (for example, alanine, valine, leucine, and isoleucine). Some amphiphilic CPPs are derived entirely from natural proteins such as pVEC, ARF (19–31). ARF (19–31) is from the N-terminal domain of the tumor suppressor gene p14ARF protein (19–31) (Johansson et al., 2008). Chimeric peptides obtained by partially covalently bonding hydrophobic fragments and NLS *via* amphiphilic CPPs, such as Pep1 (KETWWETWWTEWSQPKKRKV), MPG (GLAFLGFLGAAGSTMGAWSQPKKKRKV) are both based on the SV40 NLS (PKKRKV) (Milletti, 2012). Previous studies have shown that the same amphiphilic CPP may have different secondary structures, and their binding ability to the hydrophobic/hydrophilic interface may change under different conditions (Eiriksdottir et al., 2010). Amphiphilic CPPs, such as MAP, can interact strongly with negatively charged phospholipids, and MAP with an alpha-helical structure will spontaneously insert into the lipid monolayer. In addition, structural analysis of peptide/lipid interactions showed that MPGs with β-sheet structures are more sensitive to charge than α-helical structures (Borrelli et al., 2018).

There are relatively few numbers of hydrophobic CPPs, and their structure contains a large number of non-polar residues or only a few charged amino acids (less than 20% of the sequence). Natural hydrophobic CPPs found so far include C105Y (Rhee and Davis, 2006), Bip4 (Gomez et al., 2010), and K-FGF (Carnevale et al., 2018). Different from what is known for most amphiphilic cationic CPPs, the peptide sequence of hydrophobic CPPs does not significantly affect cell uptake (Gomez et al., 2010).

## Cellular Uptake Mechanisms of CPPs

**Figure 2:**
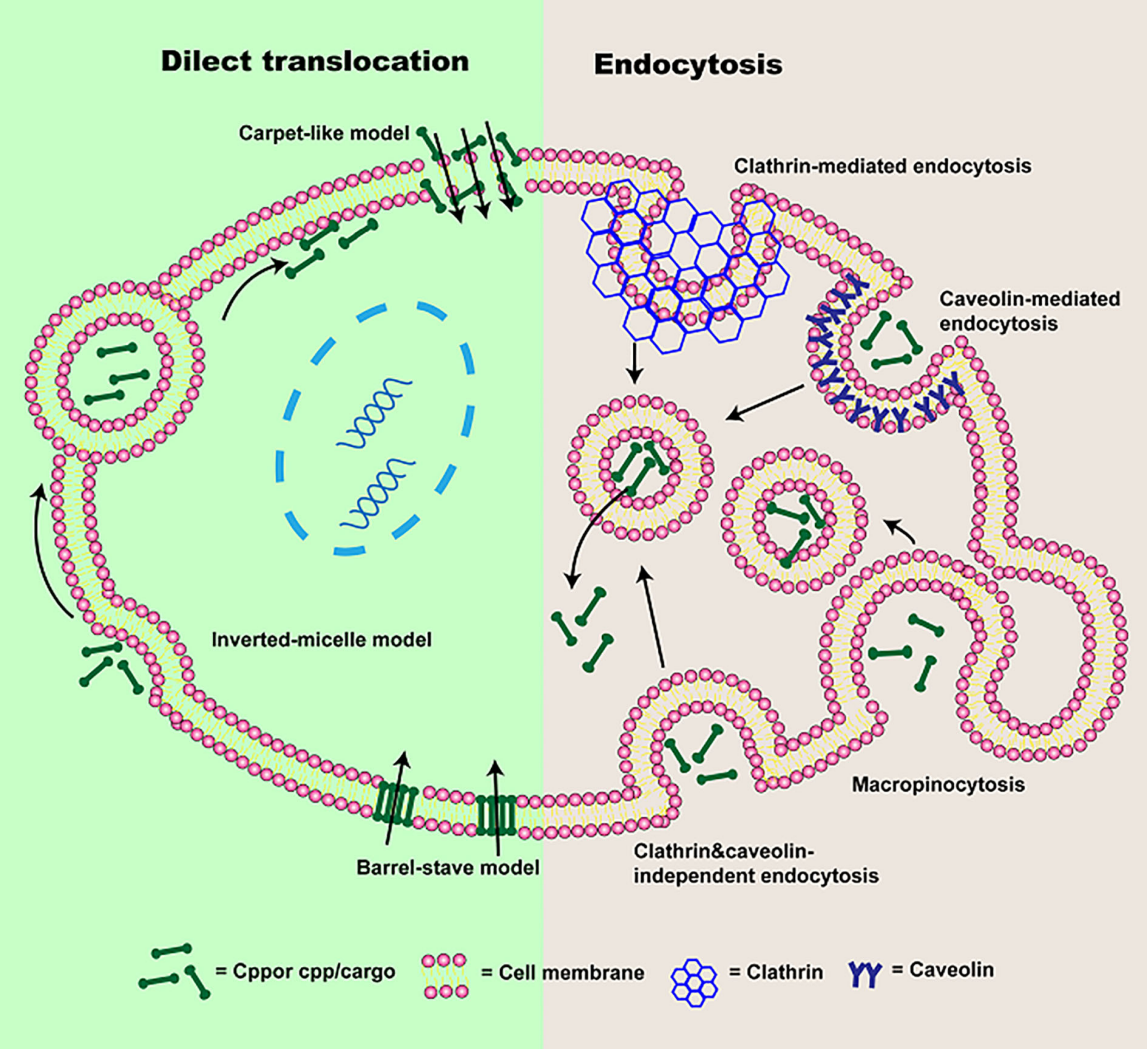
The schematic of cellular uptake mechanisms of CPP or CPP/cargo. Two types of pathways were presented: direct translocation and endocytosis. Direct translocation is divided into three models: “Barrel-Stave” model, “Carpet-like” model, and the Inverted-micelle model. Endocytosis is composed of macropinocytosis, caveolin-mediated endocytosis, clathrin-mediated endocytosis, and clathrin- and caveolin-independent endocytosis.

As we all know, it has been proven that CPPs can transport various cargoes into cells (Kristensen et al., 2016; Tashima, 2017; Derakhshankhah and Jafari, 2018). However, considerable controversies regarding the mechanism of cellular uptake still exist, which is mainly due to the properties of CPPs or transported cargoes (for instance, concentration, structure, etc.), cell types (membrane lipid composition, etc.), and the experimental conditions (such as pH and temperature) (Fretz et al., 2007; Kauffman et al., 2015; Akahoshi et al., 2016). It seems to be consensus on the internalization mechanisms of various CPPs or CPP/cargoes (Kauffman et al., 2015; Zhu and Jin, 2017; Borrelli et al., 2018; Pescina et al., 2018). The cellular uptake pathways of CPPs or CPP/cargoes have been generally divided into two types according to whether energy is required or not in the process of internalization (Chugh et al., 2010; Zhu and Jin, 2017): direct translocation and endocytosis (Tashima, 2017; Yang et al., 2019a), which will be described in this paper (**Figure 2**).

### Direct Translocation

Direct translocation, also known as the non-endocytic uptake pathway, is energy-independent (Reissmann, 2014; Tashima, 2017; Derakhshankhah and Jafari, 2018). It occurs initially through electrostatic interaction or hydrogen bonding between phospholipid bilayer and CPPs or CPP/cargoes (Zhu and Jin, 2017; Pescina et al., 2018). The interaction is followed by CPPs or CPP/cargoes entrance *via* pore formation or membrane destabilization (Guidotti et al., 2017). According to the different transduced mechanisms, direct translocation is mainly divided into three models: “Barrel-Stave” model (Munjal et al., 2017), “Carpet-like” model (Guidotti et al., 2017) and Inverted-micelle model (Islam et al., 2018), which were presented in **Figure 2**.

In “Barrel-Stave” model based on pore formation, amphipathic CPPs or CPP/cargoes insert into the cell membrane, their hydrophobic regions combine with phospholipid in the cell membrane, while their hydrophilic regions combine with the hydrophilic head of the phospholipid, forming the “barrel-like” pore, and transmembrane movements are increased (Kauffman et al., 2015; Habault and Poyet, 2019). Thereafter, CPPs are delivered into the cytoplasm (Borrelli et al., 2018). Whereas, the inverted-micelle model is dependent on the invagination of a phospholipid bilayer and the formation of the inverted micelle (Guidotti et al., 2017). In this process, electrically charged CPPs or CPP/cargoes residues binds to phospholipids on the cell surface, the hydrophobic regions of CPPs or CPP/cargoes interact with the cell membrane, forming inverted micelles (Wang et al., 2014; Islam et al., 2018; Yang et al., 2019a). And then CPPs or CPP/cargoes are transported into the cell by inverted micelles. However, it is worth mentioning that the inverted-micelle model is suitable for CPPs or CPP/cargoes with hydrophobic amino acid residues (Thennarasu et al., 2010). In the “Carpet-like” model, CPPs or CPP/cargoes are transported into the cell by charge interaction between CPPs and cell membranes (Xu et al., 2019). CPPs or CPP/cargoes cover on the cell membranes surface in a carpet-like manner, their hydrophobic part interacts with the hydrophobic region of the cell membrane (Futaki et al., 2007). Once the concentration of CPPs or CPP/cargoes is high, their hydrophobic part will be flipped by the hydrophobic core of cell membrane, the cell membrane fluidity is increased (Murray et al., 2016). Finally, the cell membrane is disrupted and CPPs or CPP/cargoes are transported into the cell (Yang et al., 2019a). However, direct translocation is most suitable for CPPs or CPPs associated with small cargo enter into the cell, while large molecules weight CPPs or CPP/cargoes mainly depend on endocytosis.

### Endocytosis

In addition to uptake by direct translocation, CPPs or CPP/cargoes can also be translocated into the cell *via* endocytosis. It has been shown that energy-dependence endocytosis is the prevailing cellular uptake mechanism for large molecules weight CPPs or CPP/cargoes (Borrelli et al., 2018; Yang et al., 2019a). So far, four different pathways including macropinocytosis (Wadia et al., 2004), caveolin-mediated endocytosis (Fittipaldi et al., 2003), clathrin-mediated endocytosis (Yang et al., 2016), and clathrin- and caveolin-independent endocytosis (Yang et al., 2019a) have been used to describe endocytosis, the schematic is also presented in **Figure 1**.

Macropinocytosis based on receptor-independent and lipid raft-dependent is preferred endocytic pathways for CPPs associated with large cargoes. Owing to the induction of growth factors and stimulation of actin, CPP/cargoes can be transferred into cells by mature vesicles (Xu et al., 2019). The Cellular uptake process through macropinocytosis as follow: firstly, CPP/cargoes interact with membrane proteoglycans to activate the rac protein in the cytoplasm. And F-actin organizations are triggered by signals from the rac protein. The actin microfilaments shrink, cell membrane deforms, protrusions, and endocytic vesicles are formed. At last, CPP/cargoes are endocytosed into cells (Pujals and Giralt, 2008). Clathrin-mediated endocytosis, also named as receptor-mediated endocytosis, is the process by which cells specifically uptake extracellular. CPP/cargoes firstly attach to receptors in the cell membrane, and curvature is produced after the interaction between epsin protein and cell membrane (Yang et al., 2019a). Subsequently, a pit is formed by recruiting clathrin and hetero-tetrameric protein (AP-2) and developed into clathrin-coated vesicles containing CPP/cargoes (Richard et al., 2005). The endosome is following formed in the cytoplasm (Yang et al., 2019a). Whereas, caveolin-mediated endocytosis is similar to clathrin-mediated endocytosis, but it is related to caveolin (Aderem and Underhill, 1999). In this process, CPP/cargoes would specifically recognize receptors on the lipid rafts that is a hydrophobic region rich in cholesterol and sphingomyelin (Zhao et al., 2015). Cavin-1 connects to caveolin, a pit is generated and invaginated with the gradual increase of the number of cavin-1 and caveolin complex. The caveolin-coated vesicle is obtained, and the endosome is formed. Additionally, clathrin- and caveolin-independent is another endocytic pathway, mainly occurs in specialized cells such as macrophages (Mcclorey and Banerjee, 2018). CPP/cargoes can be recognized and tagged by opsonins. And CPP/cargoes are attached to the Fc receptor from cell membrane and actin is stimulated, the cell membrane coated CPP/cargoes are generated and subsequently, CPP/cargoes are translocated into the cytoplasm (Yang et al., 2019a). Although energy-dependence endocytosis is the main route for CPP/cargoes entrance cells, CPP/cargoes remain coated in endosomes and difficult to exert their biological activity. Therefore, in order to avoid degradation from lysosomes, CPP/cargoes must escape from endosomes. Some reports have shown that pH gradient formation, an increasing in vesicles concentration and the attraction of differently charged endosomes membrane with CPPs all cause membrane stiffening and rupture, contributing to CPP/cargoes escape from endosomes (Borrelli et al., 2018; Xu et al., 2019). However, it is still a challenge.

## Application of CPPs in the Diagnosis and Treatment of Various Diseases

CPPs were increasingly applied in drug delivery and disease diagnosis by precise control the transmembrane transport. Membrane translocation ability of CPPs was an important element in inflammation, central nervous system disorders, ocular disorders, and cancer treatment. Basic researches were performed to identify potential application value of CPPs combined drugs. CPPs application in preclinical studies has obtained great achievements, demonstrated the boundless potential of CPPs-based therapies. Some recent studies of CPPs to deliver cargos are underway in the clinic, detailed data were listed in **Table 2**. There was not much unambiguous evidence for CPPs modified system could work as expected. It is disappointing that no CPPs-based drugs have been approved by the FDA. In this section, we reviewed the recent development of CPP’s research in the application of various diseases from the pros and cons.

**Table 2:** Examples of CPP-conjugated therapeutics under clinical development.

Compound	CPPs	Cargos	Organization	Therapeutic application	Status	Effect	ClinicalTrials.gov ID	Refs
AM-111	TAT	D-JNKI-1	Auris Medical, Inc.	Acute Inner Ear Hearing Loss	Phase 3	AM-111 exhibited effective otoprotection in idiopathic sudden sensorineural hearing loss after acute cochlear injury	NCT02561091 Completed 2017	(Staecker et al., 2019)
P28GST	P28	Glutathione-S-transferase	University Hospital, Lille	Crohn’s disease patients	Phase 2	P28GST induced slight changes of overall fecal bacterial composition in Crohn’s disease patients	NCT02281916 Completed 2018	(Capron et al., 2019)
P28	P28	non-HDM2-mediated Peptide Inhibitor of p53	Pediatric Brain Tumor Consortium	Central Nervous System Tumors	Phase 1	Data demonstrated that phase II adult recommended dose of p28 is well-tolerated for children with recurrent CNS malignancies.	NSC745104 Completed 2017	(Lulla et al., 2016)
P28	P28	P28	CDG Therapeutics, Inc.	Solid Tumors That Resist Standard Methods of Treatment	Phase 1	No Study Results Posted	NCT00914914 Completed 2017	ClinicalTrials.gov
XG-102	TAT-	dextrogyre peptide	Xigen SA	Postoperative Ocular Inflammation	Phase 3	Ocular inflammation postoperative effect of XG-102 with a single subconjunctival injection after ocular surgery was lower than dexamethasone eye drops	NCT02508337 Completed 2017	(Chiquet et al., 2017)
DTS-108	a highly charged oligopeptide of human origin	SN38	Drais Pharmaceuticals, Inc.	Tumor	Phase 1	Advanced or metastatic solid tumors patients could receive a DTS-108 dose of 313 mg/m^2^ every 2 weeks *via* intravenous. The maximum tolerated dose of DTS-108 was 416 mg/m^2^.	NA	(Coriat et al., 2016)
AVB-620	ACPPs	Cy5 and Cy7	Avelas Biosciences, Inc.	Tumor imaging	Phase 1	AVB-620 improved intraoperative cancer visualization with high safety. NOAEL in rats with single-dose was more than 110-fold of human clinical application dose.	NCT02391194 Completed 2017	(Miampamba et al., 2017; Unkart et al., 2017)

### Application of CPPs in Cancer Treatment

Up to now, researchers have developed more than 1800 kinds of CPPs to deliver cargos from basic research to clinics in therapeutics delivery, gene editing, and cell imaging (identified in the CPPsite 2.0 database). One trouble in cancer treatment was that tumor microenvironment or other barriers blocked drug delivery to tumor cells, especially in brain gliomas and pancreatic cancers. CPPs opened a new perspective to overcome a semipermeable hydrophobic barrier to realizing drugs’ effective delivery in tissue and subcellular construction.

Most CPPs had positive side chains, interacted with a high-density anionic charge on cell membranes. Different length polyarginines were widely used in drug delivery. The cationic charge density of CPPs was an important parameter to influence the transfection efficiency of cargos. Favaro et al. designed four polyarginines fused green fluorescent protein (R3-GFP-H6, R6-GFP-H6, R7-GFP-H6, R9-GFP-H6) by *E. coli* bio-produced. Differently charged polyarginine tails impacted the folding status of the GFP variants, R7-GFP-H6, R9-GFP-H6 could self-assembly form nanoparticles in Tris Dextrose buffer. At the early stages of incubation with HeLa cells, cellular uptake of unimolecular R3-GFP-H6, R6-GFP-H6, R7-GFP-H6 through CXCR4 receptor were improved by arginine residues number in a linear way. When R7-GFP-H6 assembled nanoparticles, penetrability was higher than free R7-GFP-H6. Multivalent cationic arginine increased nanoparticle uptake, multimerization can further increase the internalization effect. Free Rn-GFP-H6 was majorly endocytic by the CXCR4-dependent pathway, oligomerization status switch to receptor-independent uptake mechanisms. The internalization ability of CPPs was also related to cargos status (Favaro et al., 2018). In addition, Kadonosono et al. found that NRP1 binding also could promote CPP/PTD extravasation (Kadonosono et al., 2015). CPPs were also used to mediate cellular uptake of extracellular vesicles except for protein and nanoparticles. Extracellular vesicles were modified by poly-arginine to improve internalization by inducing active micropinocytosis, effective cellular internalization was influenced by the number of arginine residues. Hexadeca-arginine (R16) peptide modified extracellular vesicles exhibited relatively effective anti-cancer activity (Nakase et al., 2017).

**Figure 3:**
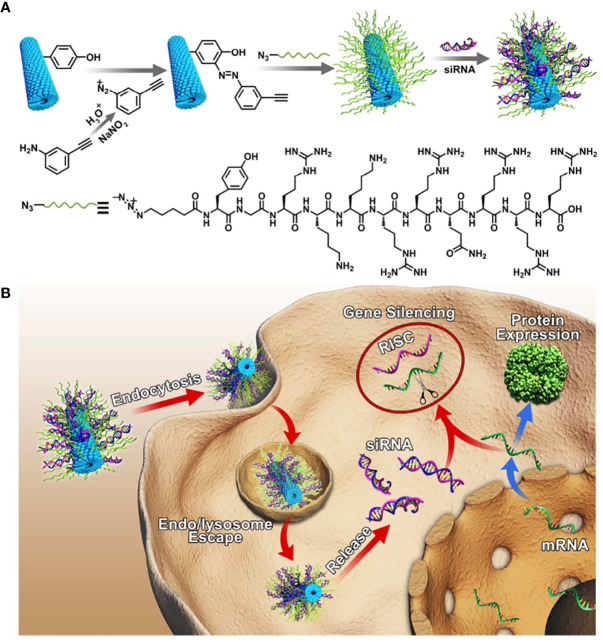
**(A)** Schematic illustration for the design of siRNA loaded TMV-TAT. **(B)** Gene silencing process of TMV-TAT within the cells (Tian et al., 2018b) (with reproduction permission).

CPPs were usually used to transfer protein by covalent bonding. However, covalent CPPs technology was not the most effective strategy to transfer macromolecules as altered biological activity and or steric hindrance (Tai and Gao, 2017). Electrostatic adsorption strategy appeared to be a preponderant approach to deliver oligonucleotide. CPPs with positive charge could efficiently condensate oligonucleotide and promote cell internalization. Tian et al. used TAT to modify the tobacco mosaic virus (TMV) to design novel siRNA carriers with high gene transfection efficiency and safety, inspired by combining viral vectors with CPPs (**Figure 3**). siRNA uptake efficiency of 45% TAT modified TMV (TMV-TAT45) was threefold than TMV in HeLa at 5 h, which benefited from not only the α-helical secondary structure but also the positive charge of TAT. TAT modified TMV endowed siRNA endosomal escape property. Gene silencing efficiency of TAT- TMV was similar to PEI25k and Lipo 2000 but showed much higher safeness. siRNA@TMV-TAT45 silenced 65% fluorescent signal of GFP expression in MHCC97-H/GFP tumors at day 10 post-injection *via* the tail vein. TMV-TAT provided a potentially safer approach to silence disease-causing genes (Tian et al., 2018b).

Generally, the linear structure of CPPs hardly obtains very gratifying transfection efficiency of oligonucleotides as low charge number, which resulted in a weak complexation and structural instability of nano-carrier. Yoo et al. synthesized a branched R9 using disulfide bonds and further constructed a novel bio-reducible cationic network using R9 as a vector (B-mR9), and branched structures provide powerful electrostatic adsorption of pDNA or siRNA. B-mR9 showed good biocompatibility and intracellular trafficking *in vitro*. In addition, B-mR9 exhibited a specifically targeted effect on the tumor by the EPR effect remaining for 48 h. B-mR9/siVEGF dramatically inhibited tumor growth by 56.5% versus control, and the therapeutic efficacy was superior to PEI25k and R9 vector in NCI-H460 bearing BALB/c nude mice model. CPPs derived cationic network provided a new approach to design gene delivery platform. Wang et al. also used Chol-based CPPs to assemble a pH-sensitive and biocompatible micelle system, which could co-deliver ULK1 siRNA and the AMPK activator narciclasine to effectively inhibit hepatocellular carcinoma in preclinical studies by regulating programmed cell death (Tai and Gao, 2017). Recent researches verified CPPs were to be the novel paradigm for siRNA oligonucleotides.

CPPs exhibited narrow clinical applications as positive charges inducing non-target and systemic toxicity. Penetrating capacity of cationic or amphiphilic CPPs was normally further powerful than neutral CPPs. Gao et al. discovered a novel highly hydrophobic cyclic CPPs (Cyclosporin A, CsA) with electronic neutral, which showed several folds higher penetrating capacity than PFV (PFVYLI) and pentapeptide VPT (VPTLQ) in MCF-7 cells, it was significantly more effective than conventional neutral CPPs. Efficiency and toxicity of cyclosporin A were compared to TAT by delivering a membrane-impenetrable pro-apoptotic peptide (PAD). When CsA conjugated to PAD, the uptake of PDA was improved 2.2- to 4.7-fold in the tumor cell lines tested by CsA, and cellular uptake of CsA-PAD was generally greater than TAT-PAD. Cytotoxicity of CsA-PAD was similar or greater than TAT-PAD in four different tumor cell lines, which depended on the cell type, but it was significantly stronger than PAD. In xenografted MCF-7 nude mice models, CsA-PAD showed comparable anti-tumor activity to TAT-PAD, but with reduced systemic toxicity. Electroneutral CPPs likely had better potential application value *in vivo* than cationic CPPs, but the accurate tissue distribution of electroneutral CPPs needs to further evaluate (Gao et al., 2017). Another strategy to reduce toxicity and non-target of positive CPPs was used polyanionic materials to coat nanoparticles, such as hyaluronic acid (HA), which was a high-affinity ligand on tumor surface-specific overexpressed marker CD44. Zhao et al. prepared a multifunctional liposome modified with TAT and HA to deliver 10-HCPT against hepatocellular carcinoma (HA/CPPs-10-HCPT-NPs), low-intensity focused ultrasound was used to precisely control drug release at tumor tissue. Zeta potential of liposome was reversed from +45.5 mV to −6.55 mV after HA modified. The penetration depth of liposome after TAT modified was improved 2.76-fold in the multicellular tumor spheroid model. Liposome combined application of HA and CPPs with the aid of ultrasound had a significantly higher tumor inhibition against hepatic carcinoma than other groups, HA-coated nano-carrier was a valuable and promising strategy for CPPs application *in vivo* (Zhao et al., 2018).

A major challenge in oncotherapy was the prognosis poor, especially for pancreatic cancer, glioma, and lymph metastasis. The curative effect of refractory tumors was a lack of significant progress because delivery systems were the inability to overcome the complex tumor microenvironment to deliver drugs reaching the treatment site. CPPs could be used as a molecular drive for cargos deep penetration of tumors. Pancreatic ductal adenocarcinoma (PDAC) had profuse collagenic fibers in the tumor stroma to resist drug penetration. Lo et al. attempted to address the target and tumor stroma penetration challenges in PDAC, two tandem peptides (pTP-PEG-iRGD and pTP-iRGD) were synthesized to prepared mixed micelle for siRNA systemic delivery. It could effectively bypass the delivery barriers of PDAC achieving tumor penetration in three-dimensional organoids and autochthonous tumors models. Furthermore, the mixed micelle complexed siRNA significantly delayed tumor growth (Lo et al., 2018). CPPs could induce cargos across the blood brain barrier (BBB) for glioma treatment. Liu et al. also prepared an anionic random-coiled polypeptide (PLG) coated CPPs (PVBLG-8) micelle to transport siRNA against glioma. PLG entangled with PVBLG-8/siRNA complex to obtain a stable structure in serum and reversed the surface potential of micelle to negative charge. In addition, micelle could respond to low pH in the tumor extracellular microenvironment to perform the cell penetration function of PVBLG-8. This carrier exhibited excellent therapeutic advantages than several commercial transfection reagents such as poly(l-lysine) (PLL) or Lipofectamine 2000 in glioblastoma tumor spheroids and U-87 MG xenograft mice model (Liu et al., 2018). In order to further improving the target limit of CPPs in glioma application, CPPs were combined with glioma-homing peptides to specifically translocate siRNA. The bonding form of Two CPPs (PF14, PF28) with targeting moistures by either covalent conjugation or non-covalent complex were optimized to increase tumor-specific targeting and gene knockdown effect. The authors established a non-covalently complexed PF14:TG1 siRNA delivery system with specificity to U87 cells showed a two-fold gene-silencing efficiency than PF14. Gene-silencing efficiency of covalently conjugated PF32 was significantly lower than PF14:TG1, due to CPPs probably shield the interaction between targeting peptide and U87 MG receptor or hinder siRNA release into the cytosol. Data demonstrated that targeting peptide non-covalently complexed CPPs was a feasible strategy for siRNA targeting delivery against the tumor (Srimanee et al., 2018). Lymph metastasis was a crucial pathway of tumor dissemination, lymph nodes nearby were the site of original tumor metastasis and further extend to the whole body. Current treatments aiming at lymphatic metastasis *via* intravenous injection were restricted non-target and poor penetration capacity due to the blood–lymph barrier. An R9 modified cabazitaxel nanoparticle (R9-CN) with 13 nm size, and the slight positive charge was verified to possess prominent lymph target and deep penetration effect after i.v. administration for potential anti-metastasis therapy. The fluorescence signal of R9-CN maintained for at least 24 h at a high level in primary tumor sites. R9-CN clearly suppressed 1.4-fold of tumor growth rate and showed a 63.3% inhibition rate of lung metastasis than CN in a breast cancer lymphatic metastasis model. CPPs modified nanoparticles were an effective anti-metastasis platform with deep lymph penetration (Hu et al., 2018).

### Application of CPPs in Inflammation

**Figure 4:**
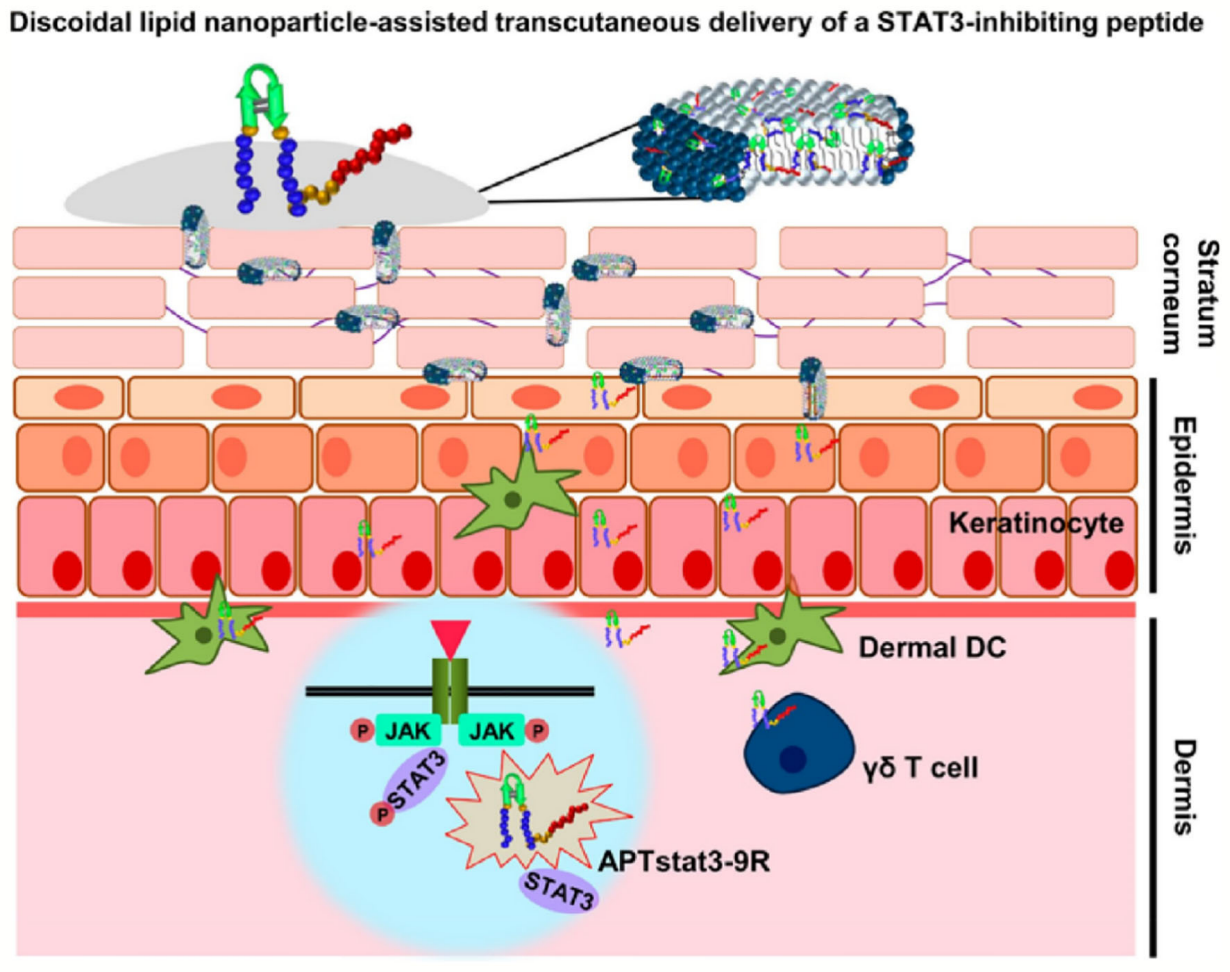
DLNPs deliver APTstat3 for Psoriasis treatment *via* transcutaneous administration (Kim et al., 2018) (with reproduction permission).

Transdermal administration is an effective means of local delivery of anti-inflammatory drugs with good compliance, stratum corneum and mucosa the main obstacles to delivery. Polyarginine peptides are commonly applied in transdermal drug delivery as efficient skin penetration ability. Gao et al. prepared lornoxicam-loaded lipid gels by R11 modified (LN-NLC-R11) to treat rat paw edema. LN-NLC-R11 significantly improved cellular uptake of NLC in HaCaT cells, remitted rat paw edema, and inhibited the inflammatory cytokines production than NLC *in vivo* (Gao et al., 2019). Due to steric hindrance, the distance between CPPs and nanoparticles will influence the efficiency of cellular internalization. The distance between CPPs and nanoparticles will influence the efficiency of cellular internalization due to steric hindrance. CPPs modified gene carriers R9Gn-chitosan/siMIF (n = 0, 4, 10) were established to inhibit pulmonary inflammation. The length of the glycine chain had little effect on the structure of the nanoparticles. Cell uptake, gene silencing efficiency, and anti-inflammatory activity *in vivo* of R9Gn-chitosan/siMIF were improved with the increasing of Gn controlled spacer arm length. R9G10-chitosan/siMIF significantly reduced the inflammation and goblet cell hyperplasia of lung tissue than R9-chitosan/siMIF in a particulate matter-induced airway inflammation mouse model (Jeong et al., 2015). Similarly, phospholipase D1 (PLD1) conjugated with TAT to enhance anti-asthmatic effect *via* intranasal administration (Lee et al., 2018). Psoriasis is a common epidermal hyperplasia disease with massive infiltration of inflammatory immune cells. Signal transducer and activator of transcription 3 (STAT3) is a key factor on the pathogenesis of psoriasis. APTstat3 is the high-affinity peptide specific to block STAT3. APTstat3 was modified by R9 (APTstat3-9R) to improve stratum corneum penetration. APTstat3-9R exhibited satisfactory results on ameliorating local psoriasis-like skin inflammation after topical treatment *via* intradermal. However, APTstat3-9R showed little skin penetrability *via* transcutaneous administration, it was blocked by the stratum corneum barrier. APTstat3-9R complexed with DMPC/DHPC to form discoidal lipid nanoparticles (DLNPs) to improve transdermal delivery with high colloidal stability (∼20 nm size) and safety. DLNPs could penetrate stratum corneum due to the lipophilicity and then move through the gaps of epidermal layers to reach dermal layers in a psoriasis-like mouse model. APTstat3-9R released from DLNPs to further act against inflammation (**Figure 4**). DLNPs effectively reduced skin edema and epidermal hyperplasia in imiquimod-induced psoriatic mice model (Kim et al., 2018). CPPs application has more advantages in the field of anti-inflammatory than anti-tumor because the local administration does not need to consider causing adverse systemic events and non-targeting.

### Application of CPPs in Central Nervous System Disorders

Central nervous system disorders mainly include stroke, Parkinson’s disease, and Alzheimer’s disease. BBB was composed of tightly connected endothelial cells without any fenestrae, which resulted in low permeability for drug brain delivery. The therapeutic effect of incurable central nervous system disorders was hardly to meet expectations due to the obstruction of BBB (Kristensen et al., 2015). CPPs transported therapeutic agents into brain *via* adsorptive-mediated transcytosis route across BBB, and they exhibited efficient BBB translocation function in submicromolar concentration without producing cytolytic effects (Sharma et al., 2016). In addition, CPPs could bypass P-glycoprotein to increase drug brain accumulation to improve therapeutic effect. However, different types of CPPs possess inconsistent BBB penetrating power. Cho et al. established culture-based multicellular BBB spheroids model with reproducible BBB functions and features to rapid screen brain-penetrating CPPs (Cho et al., 2017). Nineteen kinds of common CPPs labeled by Cy5.5 dye were investigated for their BBB penetrating ability, with DPV 15, HoxA-13, Engrailed-2, Bip(1), and Bip(2) showed the top 5 fluorescence level inside the BBB spheroids model, and all CPPs remained relatively stable except SynB1 with higher degradation rate in working media. In nude mice model, the order of CPPs brain accumulation after tail vein injection were HoxA-13, Bip(2), Bip(1), and DPV15 from high to low. However, it was not exactly consistent with the BBB spheroids model due to the difference of CPPs’ pharmacokinetics, biodistribution, and stability *in vivo* and *in vitro*. This powerful BBB model *in vitro* could serve as a valuable next generation platform to accelerate the development of central nervous system disorders therapies. CPPs’ modified strategy was a key technology to overcome BBB to enhance the therapeutic effect. The application of CPPs in Central Nervous System Disorders within the last 5 years was summarized in **Table 3** with details. In conclusion, CPPs exhibited a promising prospect for central nervous system disorders treatment through promoting BBB penetration.

**Table 3:** Recently developed on CPPs application in central nervous system disorders.

CPPs	Cargos	Delivery platform/stimuli-responsive	Disease	Model	Effect	Ref
CAMP	human metallothionein 1A (hMT1A)	Fusion protein	Parkinson’s disease	A mouse model of PD	CAMP could deliver cargo to mitochondria to alleviate mitochondrial damage	(Kang et al., 2018)
TP10	dopamine	Fusion protein	Parkinson’s disease	Preclinical animal model of PD	TP10-dopamine form accessed to the brain tissue and showed significant anti-parkinsonian activity	(Rusiecka et al., 2019)
TAT	ND-13	Fusion protein	Parkinson’s disease	Mouse model with DJ-1 knockout	TAT modified ND-13 improved the behavioral outcome and dopaminergic system dysfunction	(Finkelstein et al., 2015)
MAP	Rasagiline (RAS)	Prodrug	Parkinson’s disease	A human synucleinopathy cell model	RAS-MAP reduced protein alpha-synuclein in cells	(Vale et al., 2020)
R_9_	amyloid	Fusion protein	Alzheimer’s disease	Cellular level	Total Tau decreased	(Veloria et al., 2017)
RVG-9R	BACE1 siRNA	Chitosan-coated solid lipid nanoparticles	Alzheimer’s disease	Cellular level	Prolong residence time in the nasal cavity and improve Nose-to-brain delivery of siRNA	(Rassu et al., 2017)
K16ApoE	Curcumin	Target nanoparticles	Alzheimer’s disease	Cellular level and *in vivo* distribution	Nanoparticles could specifically accumulate in brain vasculature and also detect brain amyloid plaques.	(Ahlschwede et al., 2019a)
Penetratin	Ru(II) complex	Ru@Pen@PEG-AuNS	Alzheimer’s disease	Cellular level and *in vivo* distribution	Ru@Pen@PEG-AuNS could obviously inhibit the formation of Aβ fibrils, BBB permeability was significantly increased	(Yin et al., 2016)
R_9_	Cy5	ACPPs, ACPPs dendrimer/MMPs	Stroke	Cellular level and *in vivo* distribution	ACPPs could response MMP-2/-9	(Chen et al., 2016b)
R_9_	NA	Fusion protein	Stroke	Rat stroke model	Poly-arginine exhibited highly neuroprotective	(Meloni et al., 2015)

CPPs were effective tools for drug brain delivery against Parkinson’s disease. Kang et al. synthesized a fusion CPP using TAT and mitochondria-targeting sequence (YGRKKRRQRRRLLRAALR-KAAL) named CAMP, and used it to deliver the antioxidant protein human metallothionein 1A (hMT1A) into mitochondria to target ROS damage for preventing Parkinson’s disease (Kang et al., 2018). CAMP-hMT1A could effectively rescue movement impairment in a mouse model of Parkinson’s disease. Kim et al. constructed a kind of PEP-1-PON1 fusion protein to transduce PON1 into cells to prevent LDL and HDL oxidation induced inflammatory, oxidized-LDL level associated with Parkinson’s disease. The cellular uptake of PON1 was remarkably enhanced in neuroblastoma SH-SY5Y cells and microglial BV2 cells after PEP modified. The delivery ability of PEP-1−PON1 to cross BBB *in vivo* was evaluated after intraperitoneally injection, bio-distribution of PON1 in brain was visualized by immunohistochemistry. PEP-1−PON1 largely accumulated within the substantia nigra region of the midbrain (Kim et al., 2015). However, PON1 without CPP modified was not found to have brain delivery. PEP-1–PON1 reduced the expression of MMP-9 and protected dopaminergic neuronal against cell death in MPTP induced mice Parkinson’s disease model. Ahlschwede et al. structured a chitosan modified PLGA nanoparticles to enhance the plasma half-life and provide targeting ability to cerebrovascular amyloid deposits for Alzheimer’s disease treatment (Ahlschwede et al., 2019b). A cationic BBB-penetrating peptide (K16ApoE) was added by physical absorption onto the nanoparticles surface to generate BBB transcytosis. The plasma AUC of targeted nanoparticles was ~23 folds higher than K16ApoE-targeted nanoparticles in DutchAβ 40 treated mice. However, the brain distribution in various regions of targeted nanoparticles increased 7 to 9 times of DutchAβ 40 treated mice after K16ApoE modified. The results illuminated that K16ApoE could induce nanoparticles accumulation in the brain to reduce plasma drug concentration. K16ApoE-modified nanoparticles showed significantly greater brain uptake and also could provide specific MRI contrast to detect brain amyloid plaques. TAT was also a brain-penetrant carrier has been used in Alzheimer’s disease therapies. In addition, it has been proven TAT could bind to heparan sulfate glicosaminoglicans of extracellular cerebral deposits of amyloid to targeted treat Alzheimer’s disease (Maderna et al., 2018). MMPs were involved in neurovascular impairment after stroke, Trojan horse strategies of ACPP could be used for stroke specific detection. Chen et al. designed a gelatinase-activatable CPP to detect MMPs activity in primary neurons in culture and ischemic mouse brain *in vivo*. Cell penetrating function of R9 was shielded by anion poly-glutamate, they were conjugated by MMP-2/-9 cleaved peptide linker. Cy5-conjugated ACPP responded high expression of MMPs derived by stroke to increase cellular uptake for stroke detection. In addition, CPPs also have neuroprotective effects against stroke, such as poly-arginine and arginine-rich CPPs, efficacy was improved with the content increasing of arginine. They had the capacity to reduce neuronal calcium influx induced by glutamic acid, and the neuroprotective effect needed to induce by heparan sulfate preotoglycan-mediated endocytosis (Meloni et al., 2015). CPPs were promising tools to improve drug brain delivery for central nervous system disorders therapies.

### Application of CPPs in Otoprotective and Ocular

Acute sensorineural hearing loss mediated by traumatic has attracted more and more attention. The jun-N-terminal protein kinase (JNK) is related to cochlear inflammation. D-JNK-1 (AM-111) is an inhibitor of JNK pathway to treat acute cochlear injury. D-JNK-1 modified by TAT to realize rapid internalization by the local administration route. Phase 3 of AM-111 had completed at 2017, AM-111 indicated effective otoprotection in idiopathic sudden sensorineural hearing loss after acute cochlear injury (Eshraghi et al., 2018).

The eye consists of two parts, an anterior segment, including cornea, conjunctiva, aqueous humor, chambers, lens, and iris, a posterior segment, including vitreous humor, posterior sclera, choroid, retina. They co-formed a biological barrier to protect the eyes and simultaneously prevent drug delivery for eye treatments by several static, dynamic, and metabolic barriers. Topical application is appropriate for disease within eye anterior segment, local injection is suited to the disease within the eye posterior segment. The physiology of the eye is a challenge for ocular drug-efficient delivery, (1) The cornea is highly impervious to hydrophilic biomacromolecule, (2) the drug enters the systemic circulatory system after local injection. CPPs are potential tools for ocular drug delivery to improve bioavailability across barriers (Pescina et al., 2018). CPPs structure has an influence on the ocular distribution after topical delivery. Liu et al. evaluated several cationic CPPs on eye penetration, including TAT, polyarginine R8, polyserine S8, protamine, and penetratin. Penetratin had the optimized activity on cell uptake and ex vivo permeation (Liu et al., 2014). Chu et al. prepared an iRGD and TAT dual-modified choroidal neovascularization target nanoparticles *via* topical ocular administration. The corneal permeation of nanoparticles enhanced 5.50- and 4.56-fold after iRGD and TAT, respectively, modified. Dual-modified nanoparticles had the most cellular uptake *in vitro*, and also displayed promising targeting and penetration ability *in vivo* (Chu et al., 2017). Tai et al. developed a nano-composites to deliver antisense oligonucleotide (ASO) for gene silencing of the intraocular tumor *via* topical instillation. Polyamidoamine (PG5) was used as a gene carrier by condensing ASO, and penetratin (Pene) was introduced to improve uptake efficiency. Nanoscale PG5/ASO/Pene stably bonded by electrostatic interaction. Nano-composites displayed cell penetration and gene silencing ability after modified by Pene. PG5/ASO/Pene could also significantly inhibit the tumor volume growth in subcutaneous and orthotopic nude mice tumor models (Tai et al., 2017).

### Application of CPPs in Diabetes

At present, the commonly used method of insulin administration is a continuous subcutaneous injection by the patient. If oral insulin can be used instead of subcutaneous injection, the patient’s pain will be greatly reduced. However, insulin is unstable in the small intestine, has poor permeability in the intestinal epithelium, and low oral bioavailability. Thus, an improved insulin delivery system should be developed. Delivery of insulin using CPP is a promising candidate. In previous studies, When TAT is covalently linked to the insulin B29 Lysine residue, it can dramatically improve insulin penetration in the Caco-2 cell culture model. The bioavailability of CPP prepared insulin was six to eight times higher compared to normal insulin (Liang and Yang, 2005). Since then, including polyarginine and penetratin, and other CPPs have been seen as potential carriers of insulin. Studies by Mariko et al. Have demonstrated that the use of insulin in combination with oligoarginine (R8) can significantly increase intestinal insulin uptake without inducing observable side effects to cell (Morishita et al., 2007). In another study, Mie Kristensen et al. Found that covalently binding penetratin to insulin can increase the epithelial penetration of insulin, because penetratin is rich in Arg and Lys residues, and has a high pI value, it is positively charged at physiological pH, which promotes electrostatic interaction with the oppositely charged components on the cell surface. And they confirmed that the existence of arginine residues in the CPP sequence is a prerequisite for enhancing insulin epithelial penetration (Kristensen et al., 2015). Polyarginine can enhance insulin transmission in the rat intestinal mucosa, thereby lowering blood glucose levels, which also depends on the length of the polyarginine peptide. In the latest study, Feng Guo et al. Covalently combined amphipathic chitosan derivative (ACS) with CPP to prepare nano delivery vehicles for oral insulin delivery. The authors claim that in pharmacodynamic studies, TAT modified nanocarriers deliver insulin Significant hypoglycemic effect (40% reduction) (Guo et al., 2019). It is foreseeable that modifying the surface of nanoparticles through CPPs is a potential method to improve the absorption and delivery of insulin.

### Application of CPPs as Imaging Agents and Diagnosis

Imaging agents are essential for disease diagnosis and have the ability to trace or provide timely information on the therapeutic effect of drugs. However, it is still a great challenge to deliver imaging agents to diseased tissue due to preventing the uptake of many unnatural compounds by cell membranes (Xu et al., 2019). CPPs play a significantly important role in the delivery of imaging agents, due to their excellent permeability, high affinity, and high stability (Xu et al., 2019). Many researchers also devoted to the study of CPPs as imaging agents for diagnosis, obtaining a certain advancement in preclinical and clinical (Jiang et al., 2004; Olson et al., 2010; Miampamba et al., 2017; Unkart et al., 2017).

#### CPPs-Mediated Molecular Probes as Imaging Agents for Diagnosis

In order to accurately probe diseased sites, many CPPs-mediated molecular probes such as activatable CPP (ACPP) (Jiang et al., 2004), AVB-620 (Miampamba et al., 2017) have been studied for molecular imaging (Zhang et al., 2018a). Research has shown that ACPP can be activated by matrix metalloproteinase overexpressed in tumors and images for different enzymes by fluorescence resonance energy transfer (FRET) effect-based FI and PA agents (Jiang et al., 2004). Building on the work of ACPP, Miampamba et al. have designed and developed a novel intravenously administered AVB-620 by changing a ratiometric fluorescence readout from FRET using Cy5 and Cy7 as the couple fluorophores (Miampamba et al., 2017). AVB-620 was a fluorescent imaging agent for breast cancer diagnosis (Munjal et al., 2017). The experimental results indicated that AVB-620 can visualize the tumors under a fluorescence imaging camera system, which possesses high sensitivity and specificity in the diagnosis of lymph node status in metastatic murine breast cancer models, thus provide an excellent basis for clinical application (Miampamba et al., 2017). Moreover, AVB-620 was in the clinical phase I study in patients with breast cancer (Unkart et al., 2017). The result indicated that AVB-620 was safe, well-tolerated at doses for tumor-specific fluorescence detection which was obtained by intraoperative imaging of surgical specimens after administration of AVB-620 (Unkart et al., 2017). In addition, Zhu et al. also designed and synthesized CPPs-mediated molecular probes as imaging agents (Zhu et al., 2018). They firstly synthesized three thermally activated delayed fluorescence (TADF) chemicals (4CzIPN, NAI-DPAC, BTZ-DMAC) and TADF was loaded into amphiphilic CPPs (F6G6(rR)3R2) which was capable of self-assembling into nanoparticles in water to constructed TADF nanoparticles as imaging agents. They present low cytotoxicity and rapid membrane penetration (Zhu et al., 2018). The time-resolved luminescence imaging result indicated that more TADF nanoparticles were accumulated in cells and brighter fluorescence signals were observed in the cytoplasm with the increase of incubation time, illustrating the feasibility of TADF nanoparticles as imaging agents (Zhu et al., 2018). However, TADF nanoparticles are still basic research and expected to more researches *in vitro* or *in vivo*.

#### CPPs-Mediated Nanoplatforms as Imaging Agents for Diagnosis

**Figure 5:**
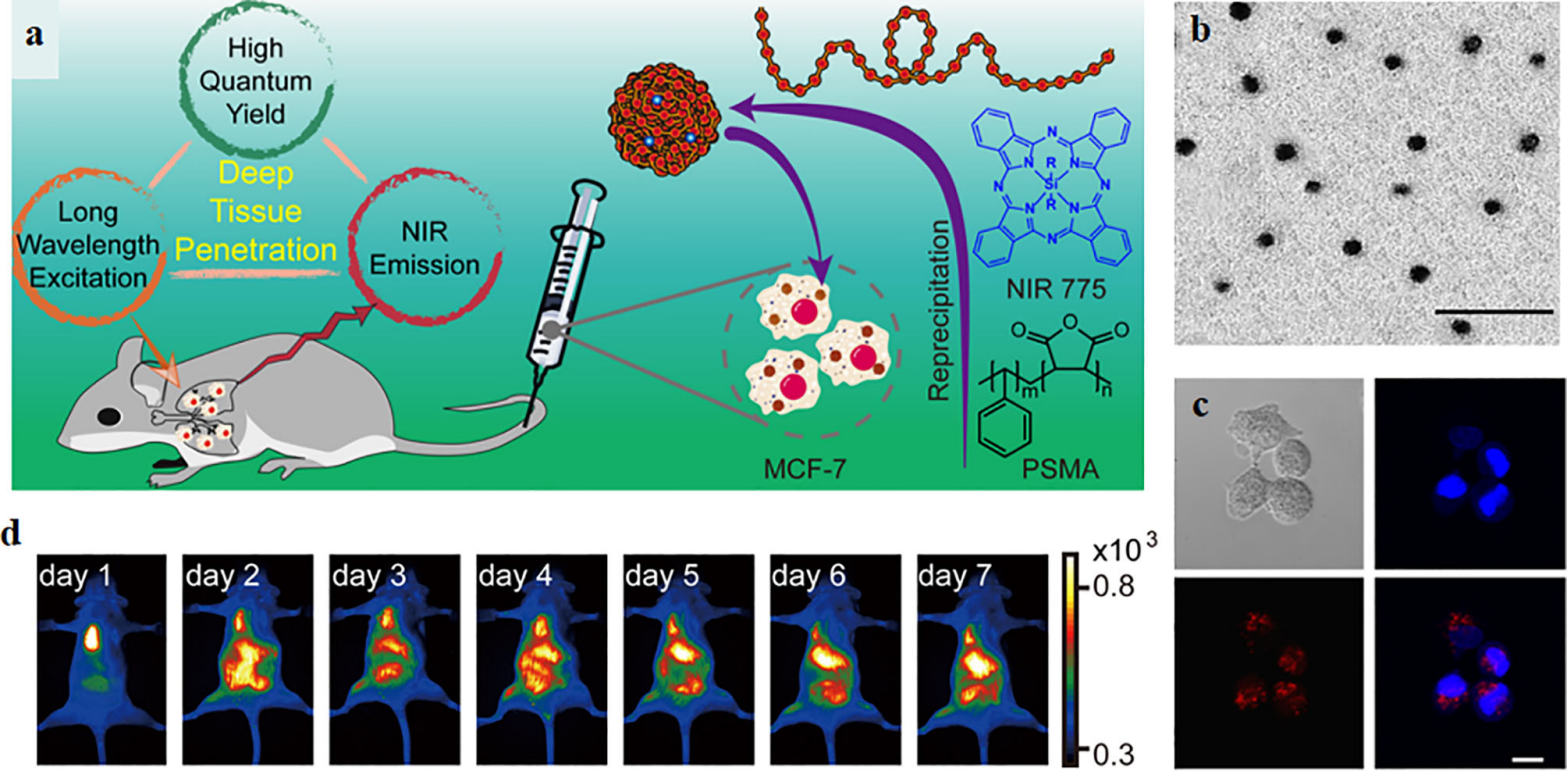
**(A)** The schematic of synthetic R8-Pdots as imaging agents for whole-body cell tracking in deep organs. **(B)** The TEM image of Pdots, the scale bar is 100 nm. **(C)** Fluorescence imaging of MCF-7 cells labeled with R8-Pdots (red) and the cell nucleus was stained by Hoechst 33258. Scale bar: 20 μm. **(D)** Fluorescence imaging of the mouse *in vivo* (Zhang et al., 2018b) (with reproduction permission).

Although organic fluorophores or fluorescent proteins have been extensively applied to imaging agents and disease diagnosis, the drawbacks of low brightness, poor stability, wide emission bandwidth limit their application (Xu et al., 2019). With the emerging of nanotechnology, CPPs-mediated nanoplatforms as imaging agents for diagnosis have attracted widespread attentions (Cai and Chen, 2007; Onoshima et al., 2015; Chen et al., 2019), this is mainly because of their advantages of low cytotoxicity, good biocompatible, high cell membrane permeability, small size, large surface areas, abundant functional group on their surface, and easily modify (Onoshima et al., 2015; Sun et al., 2017). One excellent example is CPPs-mediated quantum dots (QDs) as imaging agents for biological diagnosis (Onoshima et al., 2015; Zhang et al., 2018b; Yang et al., 2020). QDs are fluorescence semiconductor nanoparticles, possessing many advantages such as low cytotoxicity, high quantum yields, excellent stability, broad emission spectra (Cai and Chen, 2007). However, CPPs-mediated QDs not only possess the advantages of QDs, but also have high cell membrane permeability (Yang et al., 2020). Zhang et al. synthesized near-infrared semiconducting polymer dots coated with a CPP (R8-Pdots) for cell tracking in deep organs, the schematic of synthetic R8-Pdots and experimental results were presented in **Figure 5** (Zhang et al., 2018b). The particle size of R8-Pdots with good dispersion is ~12 nm. R8-Pdots presented low cytotoxicity and high cell membrane permeability for MCF-7 cells. Meanwhile, a clear and strong fluorescence signal was recorded *in vitro* and *in vivo*. The results of cell tracking capability of Pdots in live mice *in vivo* indicated that MCF-7 cells labeled with R8-Pdots were visualized in real-time, which are powerful for whole-body fluorescence imaging *in vivo* (Zhang et al., 2018b). In addition, mesoporous silica nanoparticles, superparamagnetic iron oxide (SPIO) nanoparticles, Gold nanoparticles have also been explored for imaging agents and diagnosis, on account of their low cytotoxicity and easy functionalization (Grasso et al., 2019). For example, CPPs (RGE) modified, Gd-DTPA conjugated, and doxorubicin (DOX) incorporated Fe3O4@SiO2@mSiO2 nanoparticle drug delivery system (Fe3O4@SiO2@mSiO2/DOX-(Gd-DTPA)-PEG-RGE NPs) were synthesized for MRI, explored by Gao et al. The NPs could be accumulated in U87 cells and provided a T1-T2 dual-mode contrast MR imaging result, which indicates a more accurate diagnosis (Gao et al., 2018a). Gao et al. also designed a multifunctional drug-loaded nanosystem (F/A-PLGA@DOX/SPIO) as a T2-negative contrast agent for MRI and the nanosystem realized the dynamic monitoring of DOX efficacy (Gao et al., 2018b). Although CPPs-mediated nanoplatforms as imaging agents show excellent experimental results in diagnosis, very few nanoplatforms have been translated into the clinic.

#### Radiolabeled CPPs as Imaging Agents for Diagnosis

Radiolabeled peptides as traditional imaging agents are the most widely utilized due to their high precision. Radiolabeled CPPs, as one of the peptide-mediated imaging agents, have also been developed for disease diagnosis. Recently, some radiolabeled CPPs such as [^18^F]-FPPRGD2, [^18^F]Galacto-RGD and 18F-RGD-K5 have been translated into clinical trials (Sun et al., 2017). [^18^F]Galacto-RGD was the first applied to RGD PET tracer in human, achieving high specificity and fast metabolism in cancer patients (Chen et al., 2016a; Jackson et al., 2017). And [18F]-FPPRGD2 was the first dimeric RGD peptide approved by FDA and used for humans, indicating promising outcome in glioma (Xing et al., 2014; Chen et al., 2016a). Additionally, 18F-RGD-K5 was also applied to human, and its radiation dose was determined from whole-body PET/CT. The results indicated that the radiation dose of 18F-RGD-K5 was highest in the urinary bladder wall and can be decreased by frequent voiding. Moreover, 18F-RGD-K5 could be rapidly cleared by the renal system (Doss et al., 2012).

## Limitations of CPP-Mediated Application

In the past 30 years, CPPs have been increasingly used in various disease diagnosis and therapy, as one of the most useful approaches for transfection in different cell types. CPPs have been key factors in achieving therapeutic concentrations in cells and tissue that are hard to target, consequently improving their therapeutic outcome. Their success is not only dependent on their powerful transmembrane delivery characteristic but also due to their versatility. They can be simply synthesized, modified, and improved.

However, CPPs are a double-edged sword since they may induce significant adverse effects owing to various reasons. So far, no CPP-conjugated drugs have been approved by FDA and several clinical trials have been terminated. The reasons are as follows: (1) Stability tissues *in vivo* (Pujals and Giralt, 2008). The rapid clearance from blood is a drawback as a therapeutic payload may be degraded in circulation before reaching the therapeutic site caused by enzymic degradation. (2) Immunogenicity issues. CPPs, due to its polypeptide property, will increase the risk of the undesired immune response in patients, which may not only reduce the drug effect but also cause unwanted immune stress response (Jarver et al., 2010). It can be used in high-dose and frequent intervals with the purpose of increasing exposure to reduce the immunogenicity of therapeutics, but this way results in toxicity. The other way is to inject therapeutics subcutaneously which can reduce the production of anti-drug antibodies caused by the immune system (Jauset and Beaulieu, 2019). (3) Cellular toxicity. Due to off-target cellular absorption of the therapeutics by normal tissues, CPPs can be internalized by almost all types of cells. Most research reported low toxicity of CPPs, however, it should be noticed that everything can become cytotoxic at a high concentration, and many questions need to be answered before being officially applied to the patients. The cytotoxicity of CPPs is of great concern (Saar et al., 2005). For example, the MAP, as a typical CPP, is similar in the structure of the antimicrobial lytic peptides to affect microbial cells by disturbing their plasma membranes (Zorko and Langel, 2005). It has been reported that the MAP showed a fairly high toxic effect on various cell types at concentrations higher than 1 μM through carrying out a variety of cytotoxicity assays (Aguilera et al., 2009). Due to their amphipathic effect in the presence of artificial micelles, MAPs can induce the leakage of protons, proteins, metal ions, etc., which results in cell death due to the damage of the plasma membrane (Moutal et al., 2015). (4) Low specificity. It is well accepted that cationic CPPs can bind to glycosaminoglycans (Walrant et al., 2017), but it is an unknown field whether CPPs can interact with specific membrane receptors. A widespread tissue distribution of CPP-conjugated therapeutics can reduce drug efficiency due to a lower local concentration. Therefore, maximizing specific cells targeting while ignoring normal cells is crucial. (5) Endosomal degradation after entering into cytosol. The delivery of CPPs and CPP/cargo complexes to the cytosol from endosome before lysosomal degradation is another critical problem (Erazo-Oliveras et al., 2012). It is believed that drugs remain within endosomes cannot exhibit their biological function. Therefore, CPPs should be engineered that it should promote effective endosomal escape to speed up the release of the carrier from the endosome into the cytosol. Meanwhile, the foremost important methods for delivery of CPPs are not only translocated to the target tissue or organ, but also target into specific organelles in the cell, such as nucleus and mitochondria (Horton et al., 2008), to accomplish efficient treatment (Biswas and Torchilin, 2014).

In summary, in order to realize the clinical application of CPPs, to overcome CPPs associated with treatment challenges, optimized CPPs with low toxicity, high efficiency, and specificity are urgently demanded.

## CPP-Based Optimization

As mentioned above, clinical applications of CPPs depend on the improvement of some important characters including enhancement of stability, the delay of degradation of CPPs by enzymes in the circulation, reduction of cytotoxicity, the improvement of the endosomal escape efficiency and target specificity.

### Endosomal Escape Efficiency

To date, potential mechanisms for endosomal escape have been proposed. One potential explanation is based on the positively charged CPPs, which are thought to bind to negatively charged components in the endosomal membrane (Erazo-Oliveras et al., 2012). This would lead to the formation of a membrane pore which results in the leakage of CPPs (Yang et al., 2010). Another possible reason for escape is the formation of ionic pairs between negatively charged phospholipids and positively charged CPPs (El-Sayed et al., 2009), which would partition across the endosomal membrane (Tuennemann et al., 2008).

To increase the CPPs’ release from endosome, some of the most common strategies have been used as follows. For example, the use of fusogenic lipids to improve the endosomal release of CPPs. Dioleoylphosphatidyle thanolamine (DOPE) can significantly increase the release and activity of the therapeutics from endosomes. El-Sayed et al. indicated that DOPE showed a giant improvement in transfection efficacy combining into lipoplexes or TAT-pDNA complexes. DOPE changes from lamellar phase to an inverted hexagonal phase in endosomes with lower pH levels. This transformed phase promotes the fusion of the CPP/cargo complexes and the endosomal phospholipids, which causing the destabilizing of the membrane to release the CPP/cargo complexes into the intracellular space (El-Sayed et al., 2009). The “proton sponge” effect is also used to enhance the endosomal release of CPPs. When the pH of lysosome decreases, the buffering capacity of an agent can capture a large number of protons and cause Cl-influx, which results in lysosome osmotic swelling, and finally lysosome rupture to release the internalized CPP/cargo complexes to the cytoplasm. Another commonly used agent is histidine. The imidazole group of histidine can be protonated to cause lysosome osmotic swelling and rupture of the endosomes (Beloor et al., 2015), which has been extensively used to heighten the gene expression of a TAT/pDNA complex (Lo and Wang, 2008). Another effective way is to use membrane-disruptive peptides to improve the endosomal release of CPPs (Wadia et al., 2004). As we know, viruses can overcome the endosomal trap easily, so using the mechanism of the virus to realize endosomal escape is feasible by conjugating a viral fusion sequence to CPP/cargo complexes (El-Andaloussi et al., 2006). HA2 peptide originated from the hemagglutinin protein of influenza virus is a pH-sensitive fusogenic peptide. The HA2 peptide has an α-helix structure at its N-terminus, which can be inserted into lipids. At the low pH environment inside endosomes, a conformational change exposes the α-helix structure to fuse with the endosomal lipids, resulting in the endosomal release of complexes with proteins and transportan–peptide nucleic acid (PNA) complexes in the cytosol (Trabulo et al., 2010).

### Organelle-Specific Delivery: Mitochondrial Delivery

Mitochondria are recognized as the powerhouses of cells, which control most, if not all, programmed cell death mechanisms. In the etiology of metabolic diseases, the dysfunction of mitochondria is thought to be the culprit causing some abnormalities in the patients (Szeto, 2006), such as hypertension, cancers, and some neurodegenerative diseases, seriously damage human health (Cerrato et al., 2015). However, very few therapeutic drugs have access to mitochondria (Borrelli et al., 2018). Cerrato et al. designed and synthesized a series of small novel CPPs targeting mitochondria to regulate intramitochondrial processes and enhance the biologic effects. Mitochondria-penetrating peptide (mtCPP-1) could transport 5(6)-carboxyfluorescein (5-FAM) into the plasma membrane and selectively concentrate into the mitochondria, which had no effect on mitochondrial membrane potential and inhibited reactive oxygen species release by two-fold compared with SS-31. Experimental data analysis showed the mitochondrial uptake increased by 35% compared with SS-31. No toxicity was detected even at higher concentrations. These results show that mtCPP-1 is a mitochondrial CPP. Kang et al. (2018) developed a cell-penetrating artificial mitochondria-targeting peptide (CAMP), which could conjugate the antioxidant protein human metallothionein 1A (hMT1A) to form CAMP-hMT1A successfully localized to the mitochondria. CAMP-hMT1A restored mitochondrial activity, tyrosine hydroxylase production, and inhibited ROS release after treating a cell Parkinson’s disease model. Furthermore, CAMP-hMT1A injected into the brain of the PD mouse model protected dopaminergic neuronal degeneration and movement impairment (Dinca et al., 2016).

### “Smart” Intracellular Drug Delivery Systems

**Figure 6:**
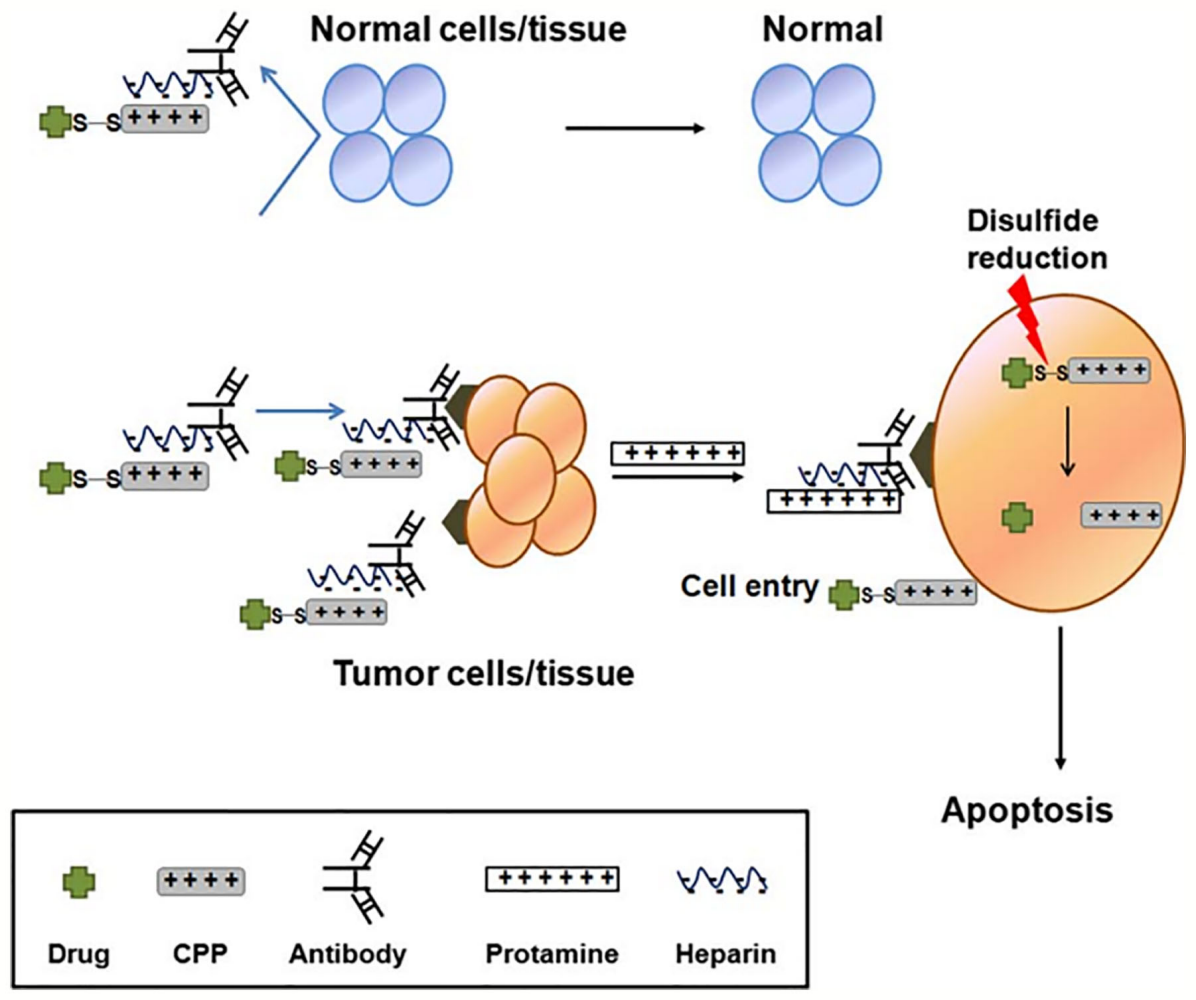
Illustration of CPP-modified ATTEMPTS system (Shin et al., 2014) (with reproduction permission).

CPPs modified drug or CPP/cargo complexes would concentrate widespread to other undesired targets because of their low targeting specificity, which leads to limited therapeutic efficiency and serious drug-induced toxic reaction. To overcome the obstacle of drug-induced systemic toxicity, a “smart” intracellular drug delivery system based on CPP was designed, named as “ATTEMPTS” (antibody-targeted triggered electrically modified prodrug type strategy) (Ye et al., 2015). The ATTEMPTS system has two components, one is the antibody targeting part which is consisting of antibodies-conjugated heparin, another is the CPPs modified drug component. The two components form a compact complex by electrostatic adsorption between cationic CPP and the anionic heparin. In fact, the electrical charge of the CPPs is neutralized by the heparin, and this could increase the plasma stability of CPP/cargo complexes against endogenous proteases. The whole process is illustrated in **Figure 6** (Shin et al., 2014). Intravenous injection administration, the antibody will carry the whole complexes to reach the target site (Huang et al., 2010). Then the clinical heparin antidote protamine sulfate is systemically injected to separate the CPP drug from its Target-Hep (Huang et al., 2013). Due to the fact that protamine has stronger heparin-binding affinity than CPPs, the CPP/cargo complexes would be released from the complex, and the complexes could translocate across the plasma membrane into the tumor cells by the activity of the CPPs (He et al., 2014).

Shin et al. (2014) successfully prepared a recombinant chimeric TAT–gelonin fusion toxin (TAT-Gel) by the genetic engineering method. TAT-Gel bound the anionic heparin by electrostatic interaction, when the protamine sulfate is systemically injected, and 75% of TAT-Gel was instantly released in 30 min, then the CPPs translocated across the plasma membrane into the tumor cells displaying substantial tumor suppression. In addition, based on the ATTEMPTS system, TAT-Gel bound the heparin-conjugated anti-CEA mAb (T84.66) by electrostatic force, which could specifically target CEA over-expressed on colorectal cancer cells. Compared to the mice administration of TAT-gel alone, TAT-gelonin/T84.66-Hep showed a significantly augmented about 58-fold target delivery (Shin et al., 2015).

### Increasing Cell Specificity Systems: Activatable CPPs

The mechanism of CPP internalization is nonspecific binding to bilayer phospholipids on the cell membrane, which severely limits the clinical application of CPPs. A possible way to enhance the specificity is achieved by the ACPP while the CPP’s cell-penetrating effect is masked by a stimulus sensitive cleavable linkers (Savariar et al., 2013), such as pH-sensitive (Tang et al., 2018), enzyme-sensitive (Yang et al., 2015), temperature-sensitive, electricity, and magnetism-sensitive or light-sensitive cleavable linker. Once in the specific tissue environment, ACPP receives external stimulation, the linker will be cleaved, and the CPPs restore its normal activity.

Enzymes possess a great variety of functions inside organisms. In pathological tissues, such as inflammation or cancer locus, the expression level of specific enzymes (Quach et al., 2014) like protease, glycosidase or esterase is usually higher compared with their concentration in normal tissues (McAtee et al., 2014). Therefore, many tumor-associated enzymes have been extensively used for enzyme-sensitive disease diagnosis and therapy because of this tissue specificity concentration gradients. the combination of CPP/cargo complexes delivery with the enzyme-triggered system not only overcome the cell permeability obstacle of the conventional delivery system, but also the selectivity obstacle of the CPP-based delivery system.

**Figure 7:**
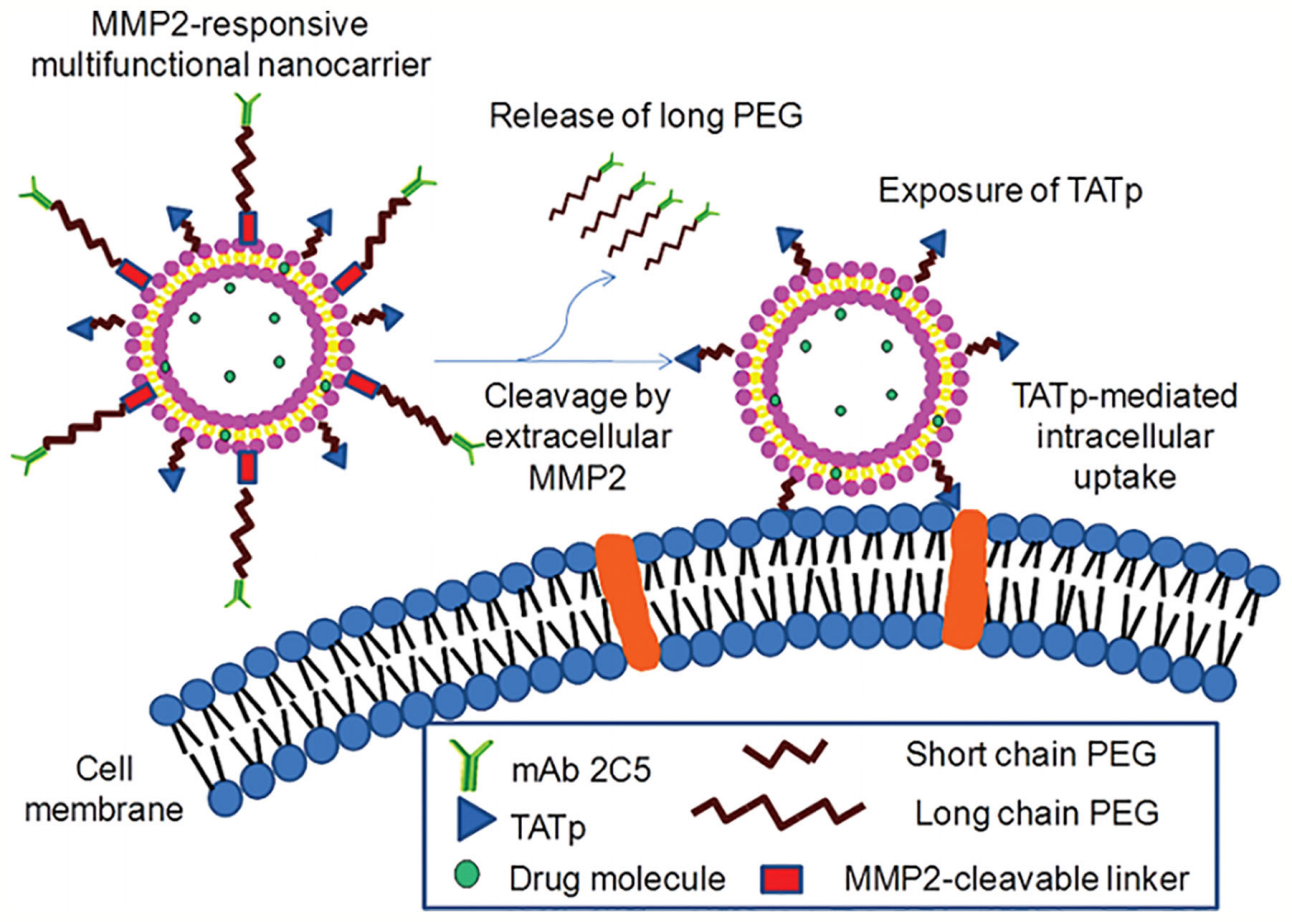
MMP2-responsive multifunctional liposomal nanocarrier and its drug delivery strategy (Zhu et al., 2012) (with reproduction permission).

The matrix metalloprotease 2 (MMP2) is overexpressed in the tumor microenvironment. Zhu et al. Designed a novel multifunctional nanocarrier, which could respond to the upregulated extracellular MMP2, enhancing tumor-specific targeting and internalization. The functionalized nanocarrier was decorated with the tumor cell-specific anti-nucleosome monoclonal antibody by using the long-chain PEG block as a steric shield for the nanocarrier (**Figure 7**). The MMP2-cleavable peptide was applied as a sensitive linker between nanoparticle lipid and long-chain PEG, and the nanoparticle was also modified with surface-attached cell-penetrating function (TATp). When the 2C5/peptide/TATp-Lip specifically targeted tumor cells, the long-chain PEG was released after the MMP2-cleavable linker was cleaved by the highly expressed extracellular MMP2, resulting in the exposure of the originally unexposed surface-attached cell-penetrating TATp, which facilitated the enhanced intracellular delivery of the system (Zhu et al., 2012).

Jiao et al. also used MMP-2–responsive peptides as the enzymatically degradable linkers to establish a gene delivery system named ch-Kn(s-s)R8-An micelles for the BBB and glioma dual-targeting. The linker conjugated angiopep-2, which can specifically bind to the low-density lipoprotein receptor-related protein-1 (LRP1) overexpressed on glioma cells. The micelles could effectively target to the glioma cells and then further migrate into the center of the tumor after the MMP2-cleavable linker was degraded by the overexpressed extracellular MMP2, resulting in the exposure of R8. This delivery system showed high gene transfection efficiency and improved uptake in glioma cells. The application of ch-K5(s-s)R8-An/Dbait with radiotherapy dramatically inhibited the tumor growth *in vitro* (Jiao et al., 2019).

pH was the most common condition to trigger CPPs activation in tumor delivery, pH-responsive anionic materials were generally used in combination. Yu et al. synthesized two kinds of polypeptide conjugated cholesterol-polyoxyethylene sorbitol oleate, (HE)5-CPSO, and (RG)5-CPSO. They were used to forming mixed micelles to deliver PTX to the brain with high tolerance. Polyanionic (HE)5 shielded the positive charge of (RG)5 to reduce non-target uptake in physiological conditions. However, the surface potential of (HE)5 reversed when they located at extracellular tumor or endosome with lower pH, the charge transition could activate (RG)5 to promote directed micellar uptake and drug release of PTX. Mixed micelles preferentially accumulated in tumor tissues and significantly inhibited 74.84% tumor growth comparing to control in a glioma mouse model (Tian et al., 2018a). Tang et al. also prepared a PEG-PLA nanoparticle to deliver PTX, which was modified by (HE)10G5R6 peptide to obtain pH-sensitive characteristics. It shared a similar strategy with the one above. (HE)10 controlled R6 screening ON/OFF through the hairpin structural transformation at different pH conditions (Tang et al., 2018).

In addition, UV-activatable CPPs are also a promising system to overcome the selectivity obstacle of the CPP-based delivery system. Hansen et al. designed a novel method to constrain the CPPs. After radiation with UV-light, the liposomes would be triggered following uptake into cells. Tat-peptide was inserted in PEG loop to endow stealth property, both termini of Tat were linked with an alkyl chain, which one terminus was anchored to the liposomal surface and another terminus was achieved by an UV-cleavable linker that endowed CPPs in a deactivated and constrained form. Once the UV-cleavable linker cracked after UV-irradiation, the CPPs would be exposed and reinstate the activation state to transport the entire liposome into cells (Hansen et al., 2012).

As mentioned above, these ACPPs possess innovation and excitement, but the activation process is generally believed irreversible and still often occurs at off-target sites instead of the target sites. To overcome the issue that ACPPs is lack of reversibility *in vivo* targeted delivery applications. The new group of ACPPs was synthesized, termed as reversibly activatable CPPs (RACPPs). RACPPs have a high response to biostimulus, and they will revert back to original form after leaving from the specific sites of activation to avoid the nonspecific uptake of ACPPs/cargo complexes after activation. Tang et al. designed a novel pH-sensitive RACPPs (HE-CPP), which used a highly pH-sensitive masking sequence linked to a CPP *via* a polyglycine linker (HE-CPP) to mask its positive charge and prevent off-target uptake of the ACPPs/cargo complexes. The polyethyleneglycol-polylactic acid (PEG-PLA) coupled with the HE-CPP sequence to compose polymer micelles (PMs-HE-CPP), which can enhance specificity and promote encapsulated paclitaxel (PTX) targeting. PTX/PMs-HE-CPP displayed reversible charge-conversion according to the surrounding pH as well as satisfactory loading capacity, encapsulated efficiency, and size distribution (Tang et al., 2018).

## Conclusions and Future Perspectives

CPPs are a hot topic for translocating drug or CPP/cargo complexes across the plasma membrane. Since the discovery of the TAT peptide in 1988, a large number of CPPs have been developed. CPPs have been widely used to deliver different kinds of therapeutics, imaging agents, and CPP/cargo complexes, including liposomes and nanoparticles, for diagnosis and treatment of several diseases. Although CPP is developing in full swing, numerous CPP-based clinical trials have been dramatically expanded. So far, in fact, no CPPs or CPP/cargo complexes have been approved by FDA. Many issues should be addressed before translating CPPs into clinics, as following: stability *in vivo*, immunogenicity, cellular toxicity, lack of specific intracellular uptake and failure to escape from endosomes. Of course, in terms of clinical applications, cost, ease of synthesis, suitable for industrial production and elimination should also be considered. In the future, the application of CPPs should be devoted to solving these issues, and numerous new CPP-based delivery systems should be evaluated. We can use strategies of fusogenic lipids, the “proton sponge” effect or membrane-disruptive peptides for the delivery of CPPs to promote efficient endosomal escape. A series small novel CPPs had been designed and synthesized to deliver not only to the target tissue or organ but also inside the specific intracellular organelles to accomplish more efficient therapy. The major obstacles to CPP-based delivery systems are the limited cell-type specificity, due to the most CPPs are uptaken by all cell types, and the short blood plasma half-life because of the presence of proteases. Novel strategies have been evaluated to improve CPPs specificity to the target site, such as the linking of CPPs with specific ligands in the form of covalent or noncovalent. The targeting ligands include an antibody, folic acid, transferrin, and RGD peptides (Xie et al., 2013; Yang et al., 2014; Zhou et al., 2014; Yang et al., 2015; Xie et al., 2016; Meng et al., 2019). The receptors of these targeted ligands are usually overexpressed in certain tumor types other than the normal tissue. In addition, “ATTEMPTS” strategy can not only improve the specificity of CPP-based delivery systems but also protect CPPs from enzyme degradation. The ACPP is another promising system to increase specificity. In this system CPPs are masked by the stimuli sensitive cleavable linker, which will be cleaved in the specific tissue environment, then the CPPs restore its normal activity, efficiently deliver CPP-based delivery systems into the cell and avoid delivery to non-targeted site.

The design of a safe, efficient, specific CPP-based delivery system as well as easy to produce and low cost have enormous potential and significant prospects in terms of clinical applications. CPPs and CPP/cargo complexes have the potential to provide more effective methods in diagnosis and treatment of human diseases, such as cancer, inflammation, central nervous system disorders, otoprotective, ocular, and diabetes. Furthermore, we strongly believe that CPP drugs or CPP/cargo complexes will enter the market within the next few years.

## Author Contributions

All the authors contributed to the writing of the manuscript.

## Conflict of Interest

The authors declare that the research was conducted in the absence of any commercial or financial relationships that could be construed as a potential conflict of interest.
